# Effect of music-guided breathing training combined with singing on pulmonary rehabilitation in COPD patients: a randomized controlled trial protocol

**DOI:** 10.3389/fpubh.2026.1833391

**Published:** 2026-08-11

**Authors:** Yajing Xue, Zixin Wang, Zishu Yuan, Xiaoying He, Cuiling Chen, Zirui Luo, Yuting Duan, Xinhua Wang

**Affiliations:** 1 The Fifth Affiliated Hospital of Guangzhou Medical University, Guangzhou, China; 2 Artemisinin Research Center, Guangzhou University of Chinese Medicine, Guangzhou, China; 3 School of Health Management, Guangzhou Medical University, Guangzhou, China; 4 Guangzhou Zihetang Shangdu Traditional Chinese Medicine Outpatient Clinic Co., Ltd, Guangzhou, China; 5 The Affiliated Traditional Chinese Medicine Hospital of Guangzhou Medical University, Guangzhou, China

**Keywords:** chronic obstructive pulmonary disease, music therapy, pulmonary rehabilitation, randomized controlled trial, singing, study protocol

## Abstract

**Background:**

Pulmonary rehabilitation (PR) is effective for improving symptoms and functional status in patients with chronic obstructive pulmonary disease (COPD), particularly respiratory training. However, its effectiveness is often compromised by poor adherence. Music therapy has increasingly being applied to COPD PR, due to its safety, low cost, and potential to motivate and enhance participation. Therefore, an integrated intervention combining music-guided breathing training and singing may represent a more appealing alternative rehabilitation strategy. This study protocol aims to evaluate whether this music-based integrated intervention is non-inferior to conventional PR therapy in terms of functional mobility, adherence and other patient-centered outcomes will be assessed as secondary or exploratory outcomes.

**Methods:**

This is a multicenter, double-arm, parallel-group, randomized controlled non-inferiority trial. We plan to recruit 76 patients with stable moderate-to-severe COPD, and randomize them in a 1:1 ratio to either the Music Therapy group (MTG) or the Conventional Pulmonary Rehabilitation group (CPRG). The MTG will receive music-guided breathing exercises and singing; the CPRG will receive non-music-guided breathing exercises and aerobic exercise. Both groups undergo interventions at a frequency of 60 min per session, 3 times per week, for 6 weeks. The primary outcome is the between-group difference in change in the 6-min walk distance (6MWD) from baseline to week 6, assessed under a non-inferiority framework. Key secondary outcomes include dyspnea severity, respiratory health status, pulmonary function, and adherence-related measures. Exploratory outcomes include arterial blood gas parameters, Traditional Chinese Medicine (TCM) syndrome assessment, patient satisfaction, safety, and the number of acute exacerbations of COPD (AECOPD) during the 1-year post-intervention follow-up. This trial protocol has been approved by the ethics committees of all participating centers.

**Discussion:**

This study aims to evaluate whether an integrated rehabilitation intervention of music-guided breathing training and singing is non-inferior to conventional PR in terms of functional outcomes for patients with COPD. Secondary outcomes, including adherence, feasibility, and patient-reported measures, will be assessed as secondary or exploratory outcomes and interpreted cautiously. The findings may help inform the development of alternative rehabilitation strategies for this population.

**Clinical trial registration:**

https://itmctr.ccebtcm.org.cn, identifier ITMCTR2026000211.

## 1 Introduction

Chronic obstructive pulmonary disease (COPD) is a chronic respiratory disorder characterized by persistent respiratory symptoms and airflow limitation. Its high prevalence and mortality rates, prolonged treatment cycles, and recurrent acute exacerbations impose a significant burden on public health systems worldwide (1). Therefore, the management goal for stable-phase COPD is to alleviate symptoms and reduce the occurrence of acute exacerbations. Pulmonary rehabilitation (PR) is recognized as a vital therapeutic approach for COPD, aimed at enhancing respiratory muscle strength, alleviating respiratory symptoms, improving physical activity tolerance, reducing complications, and enhancing quality of life in COPD patients (2–4). However, patient compliance remains low, making long-term adherence challenging and ultimately limiting therapeutic outcomes (5). Consequently, identifying engaging interventions that effectively integrate core rehabilitation principles has emerged as a key research focus.

Breathing exercises are an important component of pulmonary rehabilitation for patients with COPD, helping to improve breathing patterns, ventilatory efficiency, and respiratory muscle function (6–8). Meanwhile, the use of singing-based interventions for chronic respiratory diseases has gradually garnered attention. During singing, rapid inhalation and prolonged exhalation require coordinated activation of the diaphragm and expiratory muscles (9). Previous studies have shown that singing-based training can produce respiratory muscle training effects in patients with COPD comparable to those of conventional breathing exercises (8). Furthermore, singing-based and other music-related interventions may help improve self-reported symptoms, exercise capacity, mental health, and treatment adherence in patients with COPD (10–14).

Music can also promote rehabilitation through rhythmic cues and emotional support. Musical rhythms serve as external rhythmic cues that help regulate breathing patterns, while the enjoyment and engaging nature of music itself can also enhance patients’ motivation and engagement during repetitive training sessions. Some studies suggest that the rhythm or melody also may be a potential mechanism for alleviating breathlessness and fatigue during exercise (15). Overall, these findings provide a theoretical basis for integrating breathing training, musical cues, and singing tasks into a unified rehabilitation model, rather than treating them as separate intervention components.

Based on the above considerations, the intervention proposed in this study was designed as an integrated rehabilitation model that uses musical rhythms to assist with breath control and employs vocalization and singing exercises to train patients to prolong exhalation and coordinate breathing with vocalization. This integrated model provides a structured rehabilitation approach for patients with COPD.

Although breathing exercises, music-assisted rehabilitation, and singing interventions have each been explored to some extent among patients with chronic respiratory diseases, evidence regarding the integration of combining music-guided breathing training with singing into a structured COPD rehabilitation model remains limited. In particular, it is unclear whether such music-based integrated strategies can achieve outcomes that are non-inferior to those of conventional pulmonary rehabilitation in terms of functional outcomes.

Consequently, this randomized controlled trial aims to evaluate the efficacy of music-guided breathing training combined with singing intervention in patients with COPD. The primary objective of this study is to assess whether this integrated intervention is non-inferior to traditional pulmonary rehabilitation training in improving the 6-min walk distance. Secondary objectives include exploring its effects on lung function, dyspnea, symptom burden, patient-reported outcomes, adherence, satisfaction, and the frequency of acute exacerbations during follow-up.

## 2 Methods and analysis

### 2.1 Study design

This trial is a prospective, multicenter, double-arm, assessor-blinded, parallel-group, randomized controlled non-inferioritytrial. Eligible participants will be randomized 1:1 to either Music Therapy Group (MTG) or Conventional Pulmonary Rehabilitation Group (CPRG). Both groups will receive interventions at a frequency of 60 min per session, 3 times per week, for 6 weeks.

The primary outcome is the between-group difference change in the 6-min walk distance (6MWD) from baseline (T0) to Week 6 (T6), which will be evaluated under a non-inferiority framework. Non-primary outcomes include dyspnea severity, respiratory health status, pulmonary function, adherence-related measures, arterial blood gas parameters, Traditional Chinese Medicine (TCM) syndrome assessment, patient satisfaction, adverse events, and the number of acute exacerbations of COPD (AECOPD) during the 1-year post-intervention follow-up.

**Figure 1:**
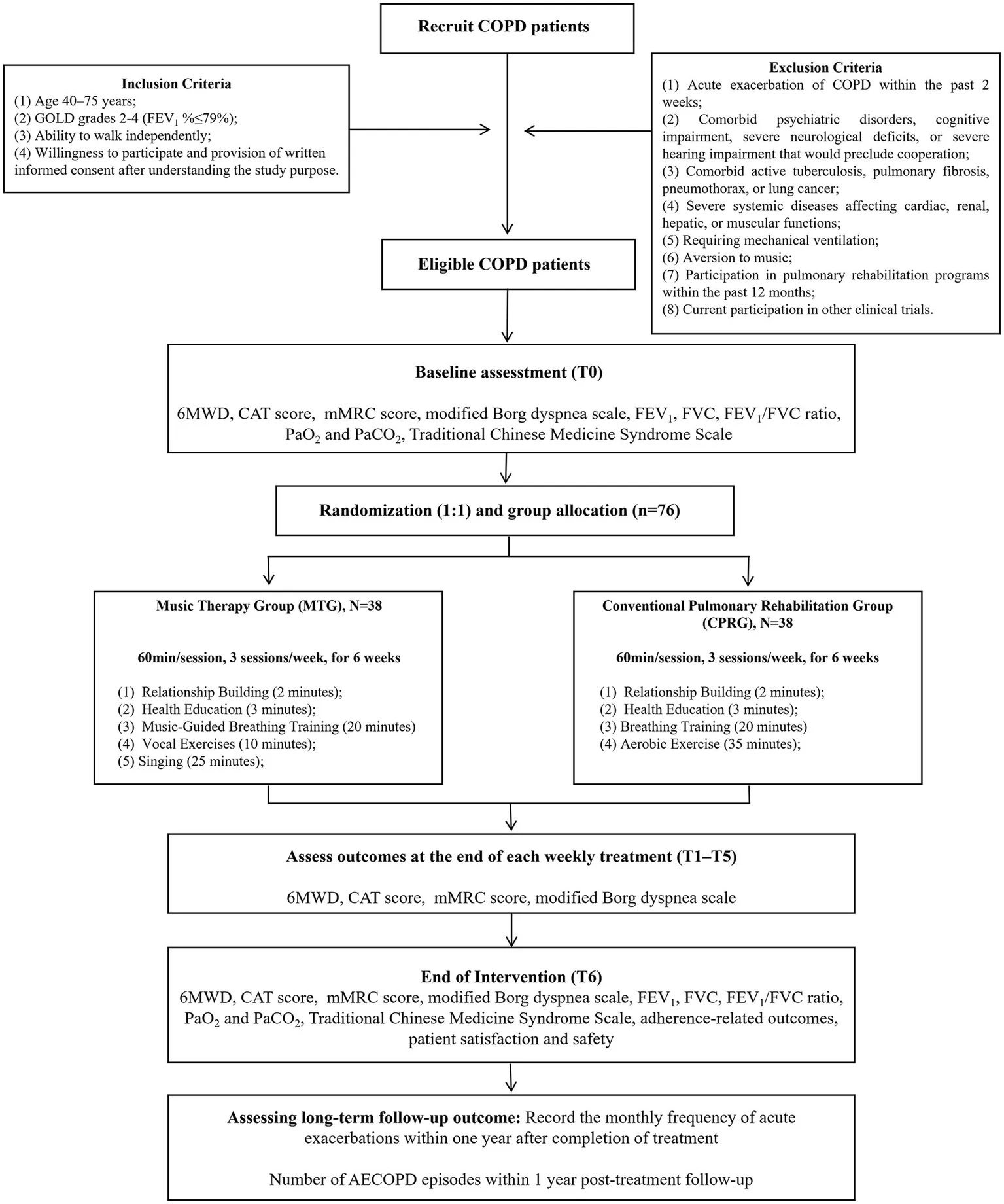
Study flowchart showing participant screening, randomization, intervention allocation, and follow-up.

The 6MWD, the COPD Assessment Test (CAT), and the modified Medical Research Council (mMRC) dyspnea scale will be measured at baseline (T0), weekly during the intervention (T1–T5), and post-intervention (T6). Pulmonary function and arterial blood gas analysis will be measured at T0 and T6. Adherence-related measures and adverse events will be recorded throughout the intervention period. AECOPD outcomes will be recorded throughout the 1-year follow-up. This study will adhere to the Consolidated Standards of Reporting Trials (CONSORT) statement for non-drug trials. The trial flowchart is shown in Figure 1, and assessment plan for primary and secondary outcome measures is shown in Table 1.

**Table 1:** Assessment plan for primary and secondary outcome measures.

Outcomes	Time points	Measurement tools and methods
Primary outcome		
Exercise capacity	T0, T1, T2, T3, T4, T5, T6	6-min walk test (6MWT)
Secondary outcomes		
Key secondary outcomes		
Dyspnea severity	T0, T1, T2, T3, T4, T5, T6	mMRC score, modified Borg dyspnea scale
respiratory health status	T0, T1, T2, T3, T4, T5, T6	CAT score
pulmonary function	T0, T6	FEV_1_, FVC, and the FEV_1_/FVC ratio
adherence-related outcomes	T1, T2, T3, T4, T5, T6	attendance records and completion records
Exploratory outcomes		
arterial blood gas parameters	T0, T6	PaO₂ and PaCO₂
Traditional Chinese Medicine (TCM) Syndromes assessment	T0, T6	TCM Syndrome Scale
patient satisfaction	T6	patient satisfaction questionnaire
safety	T1, T2, T3, T4, T5, T6	adverse event records
Long-term follow-up outcome	Monthly during the 1-year post-intervention follow-up	Number of AECOPD

### 2.2 Participants and recruitment

Recruitment for this trial will be conducted at four participating centers in China, including the Fifth Affiliated Hospital of Guangzhou Medical University, Anshun People’s Hospital, Xixiu District People’s Hospital of Anshun, and Pingba District People’s Hospital of Anshun. The study protocol has been approved by the Ethics Committees of all participating centers (Ethics Review Numbers: GYWY-L2024-96, ASSRMYYLL-20240168, 2025-EC-001, 20,250,014). Clinical staff at each subcenter will pre-screen and potential participants. To standardize procedures and advance recruitment efforts, the lead unit’s research team will hold monthly in-person or online meetings with all of the subcenter clinical members. Interested patients who meet preliminary criteria will be invited to the hospital. Researchers will provide an in-person study introduction and address patient inquiries.

Voluntarily participating patients will undergo further medical history interviews and pulmonary function tests to confirm final eligibility. After providing written informed consent, eligible patients will complete baseline assessments. The initial intervention must be administered within 14 days following the baseline assessment.

#### 2.2.1 Inclusion criteria

Patients must meet all of the following criteria for enrollment:

Diagnosis of COPD according to the 2024 GOLD guidelines, defined as the presence of chronic respiratory symptoms (dyspnea, cough, sputum production) due to airway and/or alveolar abnormalities, and/or history of exposure to risk factors, with post-bronchodilator FEV_1_/FVC < 0.7. Disease severity is classified as: GOLD grade 1 (mild, FEV_1_ ≥ 80%), GOLD grade 2 (moderate, FEV_1_ 50–79%), GOLD grade 3 (severe, FEV_1_ 30–49%), and GOLD grade 4 (very severe, FEV_1_ < 30%). ① age 40–75 years; ② GOLD grades 2–4 (FEV_1_% ≤ 79%); ③ ability to walk independently; ④willingness to participate and provision of written informed consent after understanding the study purpose.

#### 2.2.2 Exclusion criteria

Patients will be excluded if they meet any of the following criteria: ① acute exacerbation of COPD within the past 2 weeks; ② comorbid psychiatric disorders, cognitive impairment, severe neurological deficits (e.g., aphasia, agnosia), or severe hearing impairment that would preclude cooperation; ③ active tuberculosis, pulmonary fibrosis, pneumothorax, or lung cancer; ④ severe systemic diseases affecting cardiac, renal, hepatic, or muscular functions; ⑤ requirement for mechanical ventilation; ⑥ aversion to music; ⑦ participation in pulmonary rehabilitation programs within the past 12 months; or ⑧ current participation in other clinical trials.

### 2.3 Randomization and blinding

This study employed a centralized randomization system with stratified randomization by research center and variable block lengths. A computer-generated random sequence assigned eligible participants in a 1:1 ratio to either the music therapy group or the conventional pulmonary rehabilitation group. Each stratum contained 3 to 10 participants per group to ensure balanced group sizes. To conceal allocation, assignments will be placed in sequentially numbered, opaque, tamper-evident envelopes. The site coordinator will open the envelope only after the participant completes consent and baseline assessments, immediately before the first session.

Due to the nature of the interventions, participants and therapists is not blinded, while staff responsible for outcome assessment and statistical analysis will be blinded.

### 2.4 Interventions

All participants will continue their stable, regular pharmacotherapy, throughout the study, including prescribed bronchodilators and/or inhaled corticosteroids. Prior to the first treatment session, all participants will receive standardized instruction on diaphragmatic breathing and pursed-lip breathing to ensure that all groups performed these basic techniques safely and consistently. Both groups will receive treatment three 60-min sessions per week for 6 weeks. Detailed information regarding the intervention protocol, therapist training, competency requirements, progression criteria, and adherence monitoring is provided in the Supplementary material.

The Music Therapy Group (MTG) will receive a structured intervention that combines music-guided breathing exercises with singing, administered by a trained therapist following a standardized protocol. The intervention is designed to promote participants’ active control of inspiratory and expiratory muscles through coordinated breathing, voice training, and singing. Each session includs a brief introduction and status check (2 min), health education (3 min), rhythm-guided breathing training (20 min), vocalization training (10 min), and singing (25 min). Participants will first complete diaphragmatic and pursed-lip breathing exercises synchronized with a standardized piano rhythm, followed by vocalization and singing tasks. The standardized song repertoire will be selected based on principles such as steady rhythm, moderate phrase length, comfortable vocal range, and cultural familiarity. The singing sessions will progress from easy to difficult levels according to the respiratory tolerance and control performance of participants. Training intensity will be maintained within symptom-tolerable limits and monitored using the modified Borg dyspnea scale, heart rate, and peripheral oxygen saturation.

The Conventional Pulmonary Rehabilitation Group (CPRG) will receive conventional pulmonary rehabilitation interventions administered by a trained therapist following a standardized protocol. The CPRG will be matched with the MTG in terms of total intervention duration and therapist contact time. Each session will include a brief introduction and status check (2 min), health education (3 min), breathing exercises (20 min), and exercise training (35 min). Breathing exercises will include diaphragmatic breathing and pursed-lip breathing under the therapist’s guidance. Exercise training will take place in a music-free environment and include breathing exercises and the Eight Brocades. The progression of intensity and methods of physiological monitoring will be consistent with those in the MTG, but will not include any music-related therapeutic components.

The two groups were consistent in terms of intervention frequency, session duration, therapist contact time, and progress monitoring frameworks, but differed in their workload designs. The intervention group utilized rhythm-guided breathing exercises, vocalization exercises, and singing, while the control group employed breathing exercises combined with standardized exercise training. Consequently, this music-guided program was regarded as an alternative rehabilitation model rather than a supplement to traditional pulmonary rehabilitation.

To ensure consistency in the implementation of the intervention across all research centers, all therapists will undergo standardized training prior to participant enrollment. The intervention will be implemented in accordance with the protocol manual and session checklists, and the research team will periodically review protocol adherence and any deviations in implementation.

### 2.5 Outcome measures

#### 2.5.1 Primary outcomes

The primary outcome is the between-group difference change in 6MWD from baseline (T0) to Week 6 (T6). The 6MWD measurements collected weekly during the intervention period (T1–T6) will be used in supportive longitudinal data to describe the trajectory of efficacy over time, but will not serve as an additional confirmatory primary endpoint.

The 6-Minute Walk Test will be conducted according to American Thoracic Society (ATS) guidelines (16). The test will be performed on a 30-meter straight, hard-surfaced corridor, with the start and turn points marked by cone markers. Before testing, patients rest near the starting point for at least 10 min. During the test, participants will be instructed to walk as far as possible within 6 min, and slow down, stop, or rest as needed. Standardized encouragement will be provided every minute. The total distance walked within 6 min will be recorded. The modified Borg dyspnea scale will be assessed immediately before and after each test. All tests will be conducted by evaluators who have undergone standardized training, following a standardized protocol.

#### 2.5.2 Key secondary outcomes

Key secondary outcomes include: (1) dyspnea severity, assessed using the mMRC scale and modified Borg dyspnea scale; (2) respiratory health status, assessed using the CAT scale; (3) pulmonary function, including forced expiratory volume in 1 s (FEV_1_), forced vital capacity (FVC), and the FEV_1_/FVC ratio; and (4) adherence-related outcomes, including attendance rate and completion rate.

#### 2.5.3 Exploratory outcomes

Exploratory outcomes include: (1) arterial blood gas parameters, including partial pressure of arterial oxygen (PaO_2_) and partial pressure of arterial carbon dioxide (PaCO_2_); (2) Traditional Chinese Medicine (TCM) syndrome assessment, assessed using the TCM Syndrome Scale; (3) patient satisfaction; (4) safety: assessed by adverse event records; and (5) long-term follow-up outcome, defined as the number of acute exacerbations of COPD (AECOPD) episodes during the 1-year post-intervention follow-up period.

All non-primary outcome measures will be analyzed as secondary or exploratory outcome measures and interpreted with caution.

### 2.6 Data collection and management

All data are collected in the Case Report Forms (CRFs). Evaluators responsible for questionnaires and testing possessed relevant practical experience. To ensure rigorous data collection, this study will train evaluators to standardize testing principles, standardized prompts, and outcome recording protocols. To minimize measurement error, each indicator test for every subject will be administered by the same evaluator.

A unified Electronic Data Collection System (EDC) will be established. Before entering data into the EDC, all CRFs will be reviewed for errors and missing data. All data will undergo thorough verification before export to relevant statistical software. Access will be restricted to researchers and authorized personnel only.

### 2.7 Statistical analysis

All statistical analyses will be conducted in accordance with the pre-specified statistical analysis plan. The primary non-inferiority analysis will be performed simultaneously in both the intention-to-treat (ITT) and per-protocol (PP) populations.

The primary endpoint is the between-group difference change in 6MWD from baseline to week 6 (T6). The treatment effect difference will be defined as the mean change in 6MWD in the MTG minus that in the CPRG. The primary endpoint will be analyzed using a mixed model for repeated measures (MMRM), which will serve as the main confirmatory analytic approach. The model will incorporate 6MWD measurements at all pre-specified follow-up time points. Treatment group, visit time, the interaction term between group and time, study center, and baseline 6MWD will be treated as fixed effects. And an appropriate covariance structure will be used to account for the correlation of repeated measurements within participants.

Non-inferiority will be assessed based on the between-group difference in change from baseline in 6MWD at T6, as estimated by this model. If the lower limit of the two-sided 95% confidence interval for the treatment effect difference is greater than the pre-specified non-inferiority margin of −25 m, the music therapy group will be deemed non-inferior to the conventional pulmonary rehabilitation group.

Weekly repeated 6MWD measurements will also be used to support longitudinal analyses aimed at describing the trajectory of treatment effects over time; however, they will not serve as an additional confirmatory primary endpoint.

Missing data for the primary endpoint will be handled under a mixed-effects model framework based on the missing-at-random (MAR) assumption. Multiple imputation will be used as a prespecified sensitivity analysis to assess the robustness of the primary results.

For non-primary outcome measures, repeated-measure mixed-effects models will be used to analyze continuous variables, as appropriate. Continuous variables measured only once after baseline will be analyzed using analysis of covariance (ANCOVA), adjusted for baseline values, where appropriate. Categorical variables will be analyzed using chi-square tests, Fisher’s exact test, or logistic regression, as appropriate. For non-primary outcome measures, formal correction for multiple comparisons is not currently planned; therefore, all such analyses should be considered secondary or exploratory, interpreted with caution, and not treated as confirmatory evidence.

The primary non-inferiority analysis will use a one-sided *α* = 0.025; all other analyses will use two-sided tests, with *p* < 0.05 indicating statistical significance. All statistical analyses will be performed using IBM SPSS Statistics version 24.0.

### 2.8 Sample size calculation

This study is designed as a non-inferiority, and the sample size was estimated based on the between-group difference in the change in 6MWD from baseline to week 6. For the primary endpoint, the non-inferiority margin was prespecified as 25 m. This margin was established as a conservative and clinically meaningful threshold, based on previous studies indicating that the minimum clinically important difference (MID) in the 6MWD for patients with COPD is approximately 25–26 meters (17, 18). The primary hypothesis of this study is the non-inferiority of the difference in changes in 6MWD between the two groups. Secondary outcomes will be analyzed as secondary or exploratory endpoints and interpreted with caution. Given the current lack of directly comparable studies on music-guided breathing training combined with singing techniques, this study referenced standard deviation estimates of 6MWD changes (36.6 m and 32.3 m) from a similar rehabilitation intervention study as a basis for parameter selection. Under the conditions of a one-sided *α* = 0.025 and 80% power, the estimated required sample size was 60 participants (30 per group). Accounting for a 20% dropout rate, the final target sample size was set at 76 participants (38 per group).

## 3 Discussion

Enhancing long-term patient adherence while maintaining the efficacy of rehabilitation remains a key challenge in pulmonary rehabilitation for COPD. This multicenter randomized controlled trial aims to evaluate an integrated intervention combining music-guided breathing training and singing techniques, and may provide evidence for the development of more engaging rehabilitation strategies.

COPD remains a major global health challenge and continues to place a heavy burden on patients, families, and healthcare systems (19, 20). Against this backdrop, optimizing disease management remains a key priority in both clinical and public health settings. Pulmonary rehabilitation (PR) remains the cornerstone of non-pharmacological treatment for COPD, effectively alleviating dyspnea and improving physical function and quality of life (4). Existing evidence indicates that pulmonary rehabilitation is one of the most cost-effective interventions for COPD (21, 22). A meta-analysis demonstrated that pulmonary rehabilitation reduces readmission rates after discharge from acute exacerbations of COPD, alleviates dyspnea, enhances exercise endurance, and improves quality of life (23). A clinical trial further revealed that long-term sustained pulmonary rehabilitation alleviates clinical symptoms and increases 5-year survival rates (24). Conventional pulmonary rehabilitation programs primarily focus on aerobic exercise and endurance training. Aerobic and endurance training significantly enhance exercise capacity by directly targeting peripheral muscles (25). Breathing training techniques such as abdominal breathing, pursed-lip breathing, and diaphragmatic breathing effectively coordinate respiratory muscle activity and optimize thoracoabdominal movement patterns, thereby alleviating dyspnea and pulmonary hyperinflation (8). However, conventional pulmonary rehabilitation—particularly respiratory muscle training and endurance training—often suffers from poor long-term patient adherence due to repetitive content and monotonous formats (6). Consequently, exploring novel interventions that combine engaging elements with core rehabilitation principles has become a research priority.

The value of music therapy in pulmonary rehabilitation for COPD has received increasing attention in recent years (26–28). Previous studies have confirmed that group music therapy can reduce acute exacerbations and improve lung function in patients with COPD (29, 30), while a meta-analysis also indicates potential benefits in enhancing exercise tolerance and quality of life (31). Additionally, clinical studies report that music-based interventions may help patients overcome breathing difficulties and fatigue fears associated with physical activity, thereby improving their motivation to participate in pulmonary rehabilitation (32, 33). Based on this, this trial integrates music therapy with breathing exercises, using musical rhythms to guide COPD patients in performing diaphragmatic breathing and pursed-lip breathing. This approach aims to preserve the core therapeutic principles of breathing exercises while enhancing the engagement and acceptability of the intervention.

Singing is another commonly used form of music therapy. Vocal training helps patients establish more efficient breathing patterns, strengthen respiratory muscles, and improve hyperinflation and dyspnea (34–36), while the process is typically more relaxed and enjoyable (37). A recent study also found that consistent 10-week vocal training improved the 6-min walk distance in COPD patients, with efficacy comparable to conventional exercise training (38). However, previous music therapy studies predominantly focused on single interventions modalities. Based on this, the trial aims to combine music-guided breathing training with singing, to evaluate whether this integrated rehabilitation model can provide a more engaging intervention experience for COPD patients while maintaining therapeutic benefits.

Although the primary endpoint of this study is the change in 6MWD from baseline to Week 6, the repeated measurement of 6MWD during the intervention period was one of the study’s key methodological features. Compared with designs that assess outcomes only before and after the intervention, this approach allows for a more detailed description of the trajectory of functional outcomes over time. For secondary outcomes, this trial aims to investigate whether a music-based intervention, compared with conventional pulmonary rehabilitation, results in different patterns of change in outcomes related to dyspnea, health status, and physiological parameters. In particular, this study may provide further insights into the responsiveness of patient-reported outcomes (such as mMRC dyspnea scores and CAT scores) and physiological parameters (including pulmonary function and arterial blood gas parameters).

This protocol also features several design elements that may enhance its methodological value. First, this protocol uses an active-control design, with both groups receiving structured rehabilitation interventions of identical frequency and duration. This may improve comparability between groups and reduces the influence of nonspecific factors such as attention and social interaction. Second, the multicenter study framework and the use of standardized intervention and assessment procedures may help enhance the study’s feasibility, consistency, and robustness across different clinical settings.

This trial also has several limitations. First, due to the nature of the intervention, blinding of participants and intervention providers was not feasible, potentially introducing bias. However, the use of a highly objective primary outcome measure (6MWD) and strict assessor blinding maximized control over measurement bias. Second, the distribution of study centers has not yet covered more diverse geographic and economic regions nationwide, potentially limiting the generalizability of findings to some extent. Third, although repeated 6MWD assessments may provide richer longitudinal data, they may also increase the burden on participants and the complexity of study implementation. Another limitation is that, although the two groups were matched in terms of frequency, duration, therapist contact time, and monitoring framework, there was a design difference in their exercise patterns. The intervention group underwent music-guided breathing training, vocal exercises, and singing, whereas the control group underwent standardized exercise training. Therefore, the study results should be viewed as a comparison between two rehabilitation modalities, and this design difference should be taken into account when interpreting the findings.

The intervention evaluated in this study protocol is an integrated rehabilitation model, rather than a simple add-on to conventional breathing training. Its potential value lies in combining breathing training with structured, music-guided training modalities, which may support respiratory control and task engagement in patients with COPD. Therefore, this study aims to evaluate whether this integrated model is at least as effective as traditional pulmonary rehabilitation training in improving the 6-min walk distance, and to provide exploratory information on adherence, feasibility, satisfaction, and patient-reported outcomes. The results of this study may provide preliminary evidence for further evaluation of music-guided breathing training combined with singing as a viable adjunct in COPD rehabilitation.
