# Supplementary material for: Effect of music-guided breathing training combined with singing on pulmonary rehabilitation in COPD patients: a randomized controlled trial protocol
Source: Front Public Health. 2026 Aug 11;14:1833391. doi: 10.3389/fpubh.2026.1833391 (PMC13503604; doi:10.3389/fpubh.2026.1833391)
Supplement: Supplementary file 1 [file Table_1.DOCX]

**Brief Intervention Manual**

# 1. Purpose and scope

This brief public manual outlines the standardized intervention protocols for the music therapy group (MTG) and the conventional pulmonary rehabilitation group (CPRG). Its purpose is to ensure consistency in the interventions across different study sites and to clarify which components were standardized at the study protocol level.

This version is intended for public submission of journal supplementary materials. Detailed internal training scripts, site management forms, and restricted-access operational documents are retained in the internal research manual.

# 2. Common intervention principles

Both groups receive a 6-week supervised pulmonary rehabilitation program consisting of three 60-minute sessions per week. All therapists are required to complete the study’s standardized training and pass a competency assessment before interacting with the participants.

Prior to the first formal intervention, all participants receive standardized instruction in diaphragmatic breathing and pursed-lip breathing. Unless clinically indicated, participants maintain their usual medication regimen throughout the study.

Both groups follow symptom-limited progression. Training intensity was adjusted based on the Borg exertion scale, heart rate, peripheral oxygen saturation (SpO₂), and clinical observations, rather than being mechanically advanced according to a fixed schedule.

# 3. Monitoring, progression, and representative safety actions

An increase in treatment intensity should only be considered if the patient is able to complete the current treatment regimen over multiple cycles, and provided that the symptom burden remains acceptable, monitored parameters are stable, and there is no clinically significant deterioration. If tolerability is insufficient, the current treatment intensity should be maintained or reduced. The following are key safety guidelines: If blood oxygen saturation drops below acceptable levels or symptoms worsen significantly, reduce the intensity of the exercise or pause the session; if severe shortness of breath, significant hypoxia, chest discomfort, near-syncope, or clinically concerning heart rate responses occur, terminate the session in accordance with the facility’s safety procedures.

Detailed stopping thresholds and comprehensive emergency procedures are included in the internal research manual and on-site safety documents.

# 4. Fidelity across centers

To ensure consistency across all centers, all therapists follow the same structured intervention framework. The research team conducts regular quality control reviews of treatment records, monitoring indicators, decisions regarding treatment progress, and deviations from the protocol.

All sessions are documented using standardized recording forms. Any interruptions, early terminations, or deviations from the protocol must be documented, along with the reasons and actions taken.

# 5. Music therapy Group (MTG)

MTG integrates music-guided breathing training, vocal training, and singing into structured rehabilitation programs. This intervention is designed to serve as an alternative form of rehabilitation rather than an adjunct therapy; therefore, the group does not include additional aerobic exercise.

Only approved, standardized Chinese song collections may be used. Public supplementary materials disclose the hierarchical structure of the song database and its core selection principles; detailed prompt scripts remain internal training materials.

| **Component** | **Duration** | **Core content** |
| --- | --- | --- |
| Orientation and check-in | 2 min | Brief orientation, attendance confirmation, and symptom check. |
| Health education | 3 min | Brief disease-management or rehabilitation reminder. |
| Music-guided breathing training | 20 min | Standardized diaphragmatic breathing and pursed-lip breathing with rhythm cues. |
| Vocalization training | 10 min | Coordination of exhalation and phonation; avoid breath holding or excessive vocal effort. |
| Singing | 25 min | Use of a prespecified standardized Chinese song set with graded difficulty levels. |

# 6. Conventional Pulmonary Rehabilitation Group (CPRG)

The CPRG receives a standardized protocol that does not include music therapy elements. It matches the MTG in terms of the number of sessions, duration, therapist contact time, and monitoring framework to enhance the comparability of overall exposure and training load.

| **Component** | **Duration** | **Core content** |
| --- | --- | --- |
| Orientation and check-in | 2 min | Brief orientation, attendance confirmation, and symptom check. |
| Health education | 3 min | Brief disease-management or rehabilitation reminder. |
| Breathing training | 20 min | Standardized diaphragmatic breathing and pursed-lip breathing using the same core instructions as the MTG. |
| Exercise training | 35 min | Low-intensity warm-up activity, breathing-linked movement, and standardized Ba Duan Jin-based exercise. |

# 7. Standardized song bank structure

This standardized song library contains 24 Chinese songs, divided into four difficulty levels (six songs per level). To improve adherence among the target population and ensure consistency across different study sites, 12 core songs were selected from well-known patriotic songs. The remaining 12 songs serve as alternatives within the same difficulty level and can be used as substitutes as needed.

Difficulty levels are primarily determined by phrase length, vocal range, the complexity of the melodic line, tempo and rhythmic requirements, and the anticipated vocal and respiratory demands. Song progression is not automatic by session number alone; it is determined by tolerance, monitoring stability, and task-completion quality.

# 7.1 Core structure of the four graded levels

| **Level** | **Public label** | **Difficulty description** |
| --- | --- | --- |
| Level 1 | Entry level | Short phrases, narrow range, stable tempo, low vocal load. |
| Level 2 | Basic level | Slightly longer phrases, moderate contour change, still mainly mid-low range. |
| Level 3 | Intermediate level | More continuous phrasing, greater respiratory planning, and higher expressive demand. |
| Level 4 | Advanced level | Longer phrases, broader range, and greater dynamic/emotional demand. |

# 7.2 Publicly disclosed representative songs

For public supplementary purposes, To meet the need for supplementary materials, the following representative examples are provided to illustrate the structure of the graded song library: Level 1 representative songs include “Nanniwan,” “Ten Farewells to the Red Army,” and “Azalea”; Level 2 includes “Sing a Mountain Song for the Party,” “My Motherland,” and “Camel Bells”; Level 3 includes “Embroidering the Red Flag,” “Ode to the Coral,” and “On the Fields of Hope”; Level 4 includes “My Motherland and I,” “Today Is Your Birthday, China,” and “Singing Praises to the Motherland.” The complete approved song list serves as a controlled research document for the implementation of consistency management.

# 8. Abbreviations

| **Abbreviation** | **Meaning** |
| --- | --- |
| COPD | Chronic obstructive pulmonary disease |
| MTG | Music therapy group |
| CPRG | Conventional pulmonary rehabilitation group |
| SpO₂ | Peripheral oxygen saturation |
| Borg | Borg dyspnea rating |
